# Toward understanding and enhancing self-determination: a qualitative exploration with autistic adults without co-occurring intellectual disability

**DOI:** 10.3389/fpsyt.2023.1250391

**Published:** 2023-09-08

**Authors:** Sandy Thompson-Hodgetts, Jacalyn Ryan, Emily Coombs, Heather M. Brown, Adrian Xavier, Christina Devlin, Austin Lee, Adam Kedmy, Anne Borden

**Affiliations:** ^1^Department of Occupational Therapy, Faculty of Rehabilitation Medicine, University of Alberta, Edmonton, AB, Canada; ^2^Faculty of Rehabilitation Medicine, University of Alberta, Edmonton, AB, Canada; ^3^Werklund School of Education, University of Calgary, Calgary, AB, Canada; ^4^Faculty of Education, University of Alberta, Edmonton, AB, Canada

**Keywords:** autism, self-determination, autonomy, stigma, opportunity, support, adulthood, qualitative

## Abstract

**Introduction:**

Self-determination is a fundamental human right positively related to quality of life. However, Autistic people are reported to be less self-determined than non-autistic people. We aimed to (1) understand what self-determination means to Autistic people from their perspective, (2) explore their perceptions of current barriers to being self-determined, and (3) learn from Autistic people about how they would like to be supported to be self-determined.

**Methods:**

Semi-structured interviews were done with 19 Autistic adults without co-occurring intellectual disability. Data were analyzed by three Autistic and two non-autistic researchers through an iterative process of data familiarization, coding, and theme development, informed by reflexive thematic analysis. Autistic Community Partners (ACP) were also engaged throughout the study, and provided substantive feedback on all methods and results.

**Results:**

Self-determination held the same meaning for Autistic people as non-autistic people. More specifically, participants discussed having the opportunity and support to make choices and decisions in life without unnecessary control from others. Experiences of self-determination were centered around: (1) lack of opportunity, influenced by ableist expectations and discrimination, and (2) executive processing differences that interfered with choice and decision-making. Desired areas of support related to providing opportunities to (1) make choices and exert autonomy, (2) be supported to unmask and be valued as one’s authentic Autistic self, and (3) offering pragmatic support for executive processing differences.

**Conclusion:**

Autistic adults desire to be self-determined and can flourish with support, as they determine to be appropriate, which might look different from support commonly offered or sought by non-autistic people. Although individualized support was discussed, the ideal desired support was for an inclusive society that values and respects their neurodivergence, rather than imposing ableist expectations. An inclusive society is only achievable through reduced (or eliminated) stigma and prejudice against Autistic people.

## 1. Introduction

Self-determination refers to one’s ability to act as the causal agent in one’s life, to have the capacity to choose and to have choices regarding one’s quality of life free from undue external influence or interference (1–3). Self-determination is a fundamental human right, regardless of disability, as confirmed in the 2006 United Nations *Convention on the Rights of Persons with Disabilities* (4, 5) p. and ratified by Canada in 2011. Developing self-determination skills over one’s lifespan enhances quality of life and supports positive employment experiences, independent living and community inclusion, including for Autistic individuals (2, 5–7). However, Autistic individuals experience less self-determination than their non-autistic peers, including peers with other developmental disabilities (5, 8–10). There is often an assumption that Autistic people without co-occurring intellectual disability have more positive outcomes than those with co-occurring intellectual disabilities across a variety of factors associated with self-determination in adulthood (e.g., functional independence, engagement in daytime activities, participation in paid employment, quality of life) (11). However, research evaluating the relationship between self-determination and intellectual disability has mixed results (11, 12).

Why might Autistic individuals, including those without co-occurring intellectual disability, be less self-determined than others? Having the capacity to be self-determined, usually attributed to personal abilities, is necessary. Furthermore, opportunities to be self-determined, usually attributed to external factors, is vital (3). However, autism stigma and prejudice influence perceptions of capacity and opportunities to be self-determined.

The capacity to be self-determined, such as one’s knowledge and abilities to set goals, make choices, and monitor progress, as well as the ability to identify necessary supports and accommodations, or engage in supported decision-making, is essential to be self-determined (3, 13). Challenges with social-communication (based on standardized measures), depression, and differences in executive processing have been shown to predict lower self-determination in Autistic youth, including those without co-occurring intellectual disability (11, 13). Autistic people often rate their capacity higher than others (e.g., parents, teachers) (13). Discrepancies in reporting between stakeholders is common, and it remains unknown whether Autistic people overestimate their skills or others underestimate their skills (11). However, regardless of capacity, one cannot be self-determined without opportunities to do so. Having opportunities to be self-determined are also essential, yet Autistic people, including those without co-occurring intellectual disability, often lack opportunities to be self-determined across environments such as home and school (3, 11, 13, 14). Autistic people may need more support and practice to develop the skills necessary to be self-determined than non-autistic people, and evidence-based interventions to promote skills necessary for self-determination, such as those that teach self-advocacy, choice-making, goal setting and problem-solving, exist (3, 15). However, these interventions are uncommonly implemented (2, 10). Furthermore, in addition to capacity, or support for capacity, and opportunities to be self-determined in one’s daily life, societal barriers exist that may preclude self-determination more broadly.

Stigma might significantly contribute to decreased opportunities for self-determination. Autistic people commonly experience stigma and prejudice, including discriminatory attitudes and actions (16). The pervasive nature of stigma has a profound impact on the lives of Autistic individuals, extending far beyond prejudice or discriminatory attitudes. This deeply ingrained societal bias can perpetuate misconceptions, stereotypes, and misunderstandings about autism, thereby perpetuating the cycle of stigma. For instance, in high school academic settings, Autistic people may encounter lowered expectations, inadequate support, and even discrimination through overt exclusion from mainstream classrooms due to misconceptions about their abilities (17). This lack of equal educational opportunities can severely hamper their self-determination by limiting their access to knowledge, skills, and resources necessary for personal growth and success (17). Stigma can also increase camouflaging, limit social connections, and negatively influence mental and physical health (16). Autistic people may internalize stigma, decreasing their feelings of self-worth (18).

### 1.1. Objectives of the study

To our knowledge, no study to date has explored nuances of the complex array of internal (e.g., personal characteristics) and external (e.g., opportunity, stigma) factors that influence self-determination, as well as desired strategies to support self-determination, from the perspective of Autistic adults without co-occurring intellectual disability. This study aimed to (a) understand what self-determination means to Autistic people from their perspective; (b) explore their perceptions of current barriers to being self-determined, and (c) learn from Autistic people about how they would like to be supported to be self-determined.

## 2. Methods

### 2.1. Theoretical and methodological approaches

This study is situated within an interpretive constructivist approach, which aims to understand and interpret participants’ subjective experiences within an inherently complex social world (19). Within this paradigm, this work is strongly influenced by self-determination theory (SDT) (1) and the Social Model of Disability (SMoD) (20).

SDT acknowledges the influence of external regulatory mechanisms to enhance or hinder motivation, autonomy and choice. Ryan and Deci (1) suggest that three basic psychological needs must be satisfied for someone to be self-determined. The first need, autonomy, refers to having some choice or control over what happens or what one does. The second need, competence, refers to feeling capable and having a sense of accomplishment or mastery. Finally, the third need, relatedness, refers to belonging and connection to others, including support from others.

The SMoD offers a helpful critique against a deficit-oriented view of disability that was traditionally focused narrowly on physiological, anatomical or neurocognitive deficits. SMoD scholars and advocates, largely individuals who experience disability, seek liberation from identified stigma and oppression related to conceptualizations of disability and ensuing social exclusion and limiting social structures (20). They argue that disability is not located with the individual themselves but rather is “constructed” by social factors and impediments that restrict meaningful social engagement and participation and equitable access to opportunities.

This study was embedded within a larger study that utilized participatory research methods to address our research objectives specific to Autistic adults who do and do not experience co-occurring intellectual disability (21, 22). Engaging people from the Autistic community as part of the research team helped ensure that the research aligns with their needs and priorities, reduces translational barriers, and aims to disrupt ableism that has, historically, been prominent in autism research (23, 24). Autistic (*n* = 3) and non-autistic (*n* = 2) researchers and additional team members from the Autistic community (*n* = 4; hereafter called ‘Autistic Community Partners’) were partners throughout the research process, from conceptualization to dissemination. Autistic Community Partners (ACP) met monthly with core research team members. They collaborated in designing the interview guide and recruitment strategies and throughout data analysis, interpretation, and dissemination. The ACP were fairly compensated for their time, as recommended by Nicolaidis and colleagues (22).

### 2.2. Positionality of the research team

Our team comprised three Autistic and two non-autistic researchers. STH is a non-autistic ally with over 20 years of clinical and research experience related to autism. JR is an Autistic Ph.D. candidate studying the self-determination of Autistic adults with intellectual disabilities. EC is a graduate student in counseling psychology and identifies as an Autistic lesbian woman. HB is an Autistic professor who researches thriving and belonging for Autistic people. AX is a non-autistic educator with more than 15 years of experience working with diverse groups of child and adult learners. Our team also included a robust team of Autistic community partners from diverse educational and demographic backgrounds. CD is an Autistic Registered Social Service Worker finishing a second Bachelor’s degree in Disability Studies and Psychology. AL is an Autistic university alumnus with a Bachelor’s degree in Computer Science. AK is an Autistic person, university student, and supporter for other Autistic people. AB is an Autistic person, a parent of an Autistic child, and an advocate for child and disability rights for people of all abilities. Given the researchers’ diverse identities, the team co-analyzed all data and engaged in multiple discussions about potential biases and assumptions that may emerge due to their lived experiences. This process provided a system of peer examination that was crucial in making decisions on how to organize best and present the data, as well as provide relevant recommendations for improvement.

### 2.3. Inclusion criteria and recruitment

Ethical approval was obtained from the University of Alberta. All participants provided informed consent online and again verbally at the start of the interview. Inclusion criteria were (1) 18 years and older, (2) identifying as Autistic, including those who were diagnosed by a professional(s) and/or those who self-identify, (3) without a co-occurring intellectual disability (self-report), and (4) ability to complete an interview verbally or through text in English. Potential participants were recruited through email listservs and research recruitment webpages of autism support organizations in Alberta and through social media channels (e.g., Facebook pages) intended for the Autistic community (including open pages and closed membership for Autistic people). We aimed for a sample size of approximately 20 participants, which the team agreed would likely allow us to achieve data adequacy (25, 26).

### 2.4. Data collection

Semi-structured interviews, 30–60 min long, were done using Zoom (*n* = 17; camera on: *n* = 16; camera off: *n* = 1), telephone (*n* = 1), or email correspondence (*n* = 1). Example questions from the interview guide are provided in Table 1. Questions addressed all three psychological needs outlined in self-determination theory, but focused more autonomy than on competence and relatedness because Ryan and Deci noted that support for autonomy often supports increased satisfaction for competence and relatedness (1). Based on our pilot interviews and feedback from our ACP, varying options for language were provided in the interview guide to increase clarity, including the option to use the broad term ‘self-determination’ or the terms ‘choices’ and ‘decisions’ as deemed appropriate in each interview.

**Table 1:** Semi-structured interview guide.

Draft interview questions
Tell me about why you decided to participate in this study? Do you get to make choices in your day? What kind of choices are important to you? Tell me about times when having a choice is not important to you. What does self-determination mean to you? What kind of support, if any, do you need/like for making choices or decisions/to be self-determined? Are there people in your life who let you make choices/be self-determined? Are there people in your life who do not let you make choices/be self-determined? Do people take your feelings/ideas/opinions seriously? Do you feel accepted by the people around you?

Given the opportunity of an Autistic or non-autistic interviewer, 60% of participants chose an Autistic interviewer, and 40% of participants had no preference (thus were interviewed by a non-autistic interviewer). Interviews were recorded and automatically transcribed through Zoom (*n* = 17; checked for accuracy against audio recording and cleaned when necessary), recorded with a digital audio recorder and transcribed manually (*n* = 1 telephone interview), or used text provided (email). All transcriptions were anonymized by removing names of people and organizations. All participants were from Canada or the United States for the feasibility of timing and provision of gift cards. A pseudonym was chosen by or assigned to each participant.

### 2.5. Data analyses

Once transcribed, data were analyzed by three Autistic and two non-autistic researchers through an iterative process of data familiarization, coding, and theme development, informed by reflexive thematic analysis, which is appropriate for research that desires to (1) create actionable outcomes, and (2) situate experiences in broader socio-cultural contexts (27).

Initial coding was an iterative process done in multiple stages. All transcripts were reviewed by four core research team members (STH, JR, EC, AX) to address research question #1 (What does self-determination mean to Autistic adults without co-occurring intellectual disability?). To address the second (barriers to self-determination) and third (desired ways to be supported) research questions, three team members (STH, EC, AX) coded all data using a color-coded spreadsheet. Then, two team members (JR, STH) embarked on initial theme development, informed by the basic psychological needs put forth in self-determination theory: autonomy (self-directed freedom), competence (confidence in one’s ability), and relatedness (trusting and respectful relationships) (1). All codes from the spreadsheet were physically printed, and two team members used mind mapping to explore and understand relationships between ideas (28). Themes and subthemes underwent multiple iterations by the core research team and ACP. Numerous data excerpts supported the final theme and sub-theme development.

Rigor was demonstrated by established methods of trustworthiness and authenticity, including: reflexive journaling and dialog between team members, prolonged engagement by team members immersed in autism research, interdisciplinary team composition, and engagement of team members with lived experience (29).

## 3. Results

Participants included 19 Autistic adults (mean age = 34.8 years, range 18–62 years) who represented diversity across many demographic variables. See Table 2 for a summary of participant demographics.

**Table 2:** Participant demographics.

Age (years)	
Mean	34.8
Range	18–62
Identified gender (*n*)	
Woman	6
Man	6
Non-binary	1
Transfeminine	1
Autigender	2
No answer	3
Geographic location (*n*)	
Canada	11
USA	8
Who diagnosed? (*n*)	
Self-diagnosis	3
Health care provider	16
Educational attainment (*n*)	
High school	2
Current college student	2
Current university student	3
College diploma	3
Undergraduate degree	4
Graduate degree	5
Employment status (all that apply; *n*)	
Not employed	9
Employed full time (35–40 h/week)	3
Employed part time (20–30 h/week)	4
Full-time student	5
Part-time student	2
Living situation (*n*)	
On own	8
With others	11
Relationship status (*n*)	
Single	11
Partnered	6
Prefer not to say	2
Does your income meet your needs? (*n*)	
Not enough	5
Just enough	9
More than enough	5
Preferred terminology (n)	
Autistic person	13
Person on the autism spectrum	3
Person with autism	1
No preference	2

**Figure 1:**
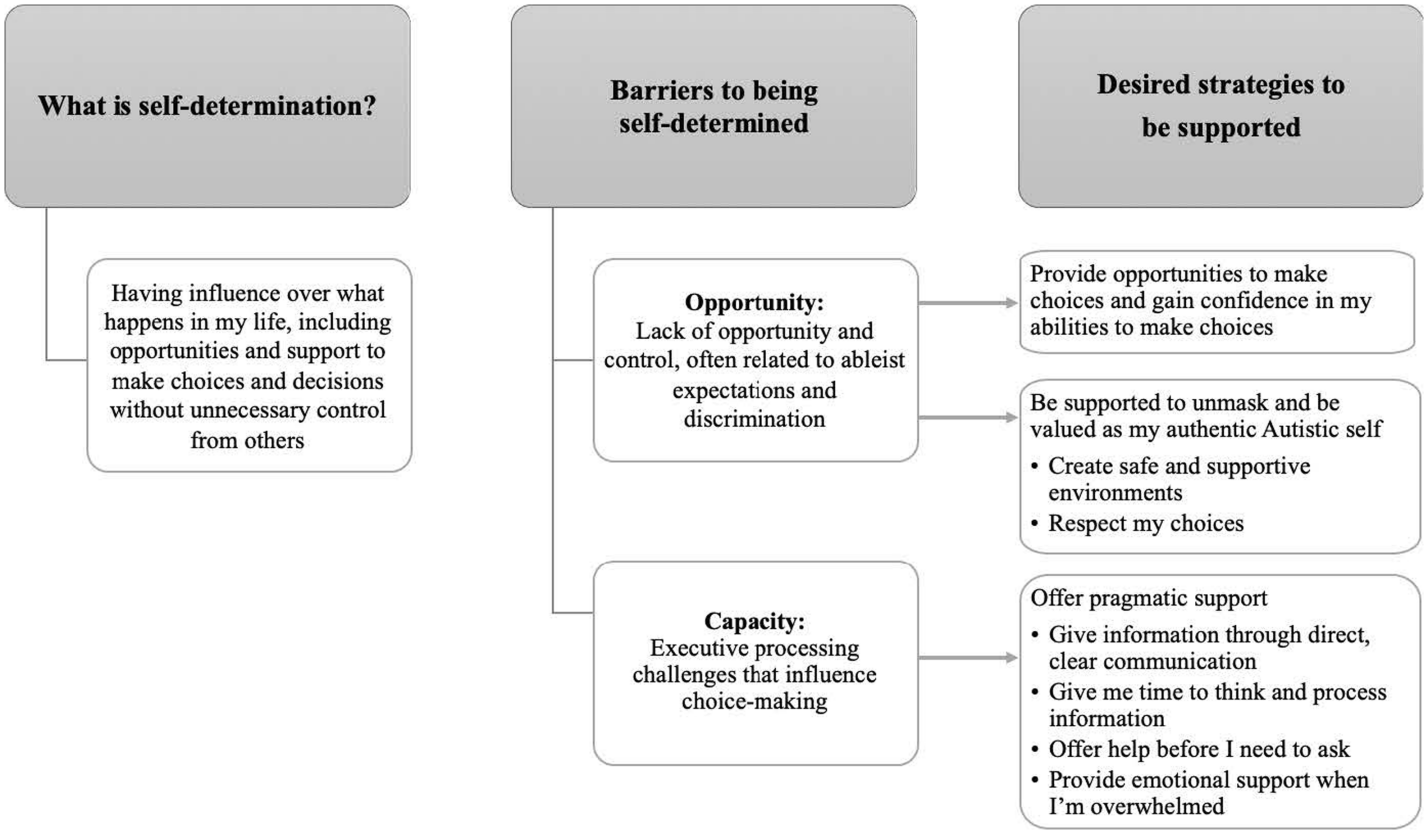
Summary of qualitative analysis.

Findings from our qualitative analyses, including conceptualizations of self-determination and developed themes related to barriers to self-determination and desired ways to be supported to be self-determined, are summarized in Figure 1 and described in detail below.

### 3.1. What does self-determination mean to autistic adults?

Self-determination held the same meaning for Autistic people as non-autistic people. More specifically, participants discussed *having influence over what happens in their lives, including opportunities and support to make choices and decisions without unnecessary control from others*. For example, Freda passionately stated, “I’m in charge of me…everything that has to do with me. Choices about my own agency…and I include in that pleasure and wanting to be who I want to be, like presenting my true self.” Kyle felt self-determination allowed him to flourish because it “is one path that allows a certain level of freedom or opportunities,” and Stephen commented that it was essential to “actively participate in life, not just exist.” Paul defined self-determination as:

…a combination of a person's drive and ability to make choices. One who has a lot of self-determination is very passionate about the things they do and are able to make clear choices regarding their life…the ability to choose to do the things necessary for a happy, healthy life, but still maintaining success in their other affairs. It's a sense of functioning independently. This doesn't mean they need to do everything alone, but they are capable of functioning as an individual in their own way.

Multiple participants discussed how self-determination co-existed with meaningful relationships and partnerships, including family and parenting responsibilities. Although Kyle affirmed the value of freedom in making choices and decisions, he also acknowledged that interdependence is important because “having a group to lift you up or care for you is important for health and wellness.” Veronica acknowledged that “of course, I work with my husband for our finances and where we want to live and that kind of stuff, but day-to-day I’m pretty free to do whatever I want … [and] being able to plan what happens to me during the day is important to me.” Nancy, a married mother of four younger children, indicated that she adores parenting, but it required significant compromise:

I get to make choices about my day-to-day stuff but it feels like those choices … [sighs] kind of get compressed when the kids get home… it’s chaotic for even neurotypical people, but when you layer in, y’know, your obligation to your kids and also your own neurodivergence, it’s a very hard balance.

When asked about what choices in life were most important, participants highlighted a variety of choices where they felt having autonomy was critical, including daily tasks such as sleep schedules and how one spends their free time and/or money, as well as longer-term choices such as “what I want to do with my life” (Stephen). Some participants, like Emma, felt “All of them…what I do with my time, what I spend my money on, where I go, all of that. It kind of determines how your life will go when you make those decisions.” Notably, although no prompts were given related to specific areas in which participants wanted to feel a sense of autonomy, eight out of 19 participants commented that control over food was important. For example, Nayeli stated “Stuff around food! I have a lot of taste and texture stuff going on, so being able to choose what I can and cannot eat, that’s important.” Similarly, Dani commented that “It’s definitely nice having control over what I eat…I like having control over my food.” Furthermore, seven out of 19 participants commented that medical autonomy was essential. For example, Tia stated, “I want more medical autonomy, including being able to control the sharing of my information.”

Overall, most, but not all, participants felt that they had opportunities to be self-determined in some areas of their lives. However, all participants also expressed barriers to being self-determined related to their experiences as an Autistic person.

### 3.2. Barriers to being self-determined

Barriers to being self-determined crossed all three psychological needs outlined in Deci and Ryan’s (1) self-determination theory, centered around both opportunity for and capacity to be self-determined. More specifically, experiences of self-determination were centred around: (1) lack of opportunity and autonomy, often related to ableist expectations and discrimination (autonomy, relatedness), and (2) executive processing differences that influenced choice-making (competence).

#### 3.2.1. Theme 1: self-determination is thwarted by lack of opportunity and discrimination

While the participants in this study tended to define self-determination in the same way that non-autistic people do, most (*n* = 14) participants discussed experiences, past and present, of limited opportunities to be self-determined. Many of the factors that contributed to these limited opportunities were externally imposed upon our participants, based on ableist expectations for the choices one makes and discrimination related to the needs and abilities of Autistic people. Marcy’s comment, which reflected comments by other participants, reinforces these experiences:

I was expected to fit into a certain box and I wasn’t given the choice to have anything different … My needs that I would try to speak up for were very quickly taught that they weren’t acceptable needs to have, they were above and beyond …those lack of choices hurt me the most when I don’t get to say ‘hey wait, no. There’s a person in here that is being deprived of a need’. If it was somebody else… nobody would be acting this way. If the lights are too bright and I’m asking for them to be not as bright, that's no different to me as somebody who's hungry and asking for food. But, to other people it’s ‘oh, you’re just being spoiled. You just want it your way. It’s not that big of a deal, you should just deal with it’.

Some participants felt that they were thwarted opportunities to be self-determined across all areas of life. For example, Nayeli expressed that “society and its push to make people like me fit in limits and does not let me make choices.” Veronica stated, “neuro-typicals like to limit us because they cannot see what we are capable of. We’re just trying to live our best lives and neuro-typicals keep telling us to stop.” Other participants felt that they lived relatively autonomous lives as adults, but still expressed a lack of opportunity in daily choices, described as “limited options” (Tia), that “available choices seem constrained, limited, or come with predetermined outcomes” (Callie), or “not getting much of a say in how it looks or what it could look like… only related to already established routines” (Marcy). The lack of opportunity to be self-determined was frustrating for participants. For example, Emma expressed frustration at the lack of being given the opportunity to do daily activities that she knew she was very capable of doing, “I cannot choose what to eat … or how to spend my time, I’m, like’Oh, I want to do this, but I cannot.’ It’s very stressful.” Kawhi reflected on negative experiences with self-determination when he was younger that strongly influenced his desire for autonomy in his current life,

If the choice concerns me, I should have a part of it and I feel frustrated if it does not … it’s the idea someone thought they could speak for me, neglecting the fact that I had my own voice and neglecting the fact that I have the ability to advocate and speak for myself. I see this thing where it’s simply just disrespectful if someone does that … it’s different if it’s consensual but it’s also something where it almost makes you feel lesser because that person sees it …that they can speak for you, even if they may not actually know you best.

The negative judgments of others related to participants’ desires and preferences often led them to doubt their ability to make appropriate choices. In particular, four participants reflected that the lack of opportunities to be self-determined contributed to these doubts. For example, Kanti stated, “I was never really allowed to be an independent person, so I’m not a very secure person in my own judgment. Often there’s a lot of imposter syndrome and such, so I’ll fret about whether I’m making the right decisions.” This sentiment was also echoed by Nayeli who said, “On one hand, making choices allows me to say ‘no’, but on the other hand, it can be anxiety provoking because I worry about making the wrong decision.” The impact of restricted opportunities earlier in life on the confidence for autonomous decision-making can be clearly seen in Marcy’s continued reflection, “now I feel like I have to have permission to do things … and I’m afraid if I just go forward …and do whatever I think to do, that I’m going to get in trouble for it, or misunderstand what I was supposed to do …” Their repeated experiences of being denied opportunity, including during their childhood, led to chronic feelings of self-doubt as shown by Marcy’s next words, “So, with everything that I choose to do [as an adult], there’s always that voice in the back of my head that’s [saying], ‘What if you are choosing wrong? What if this is going to end up going badly for reasons that you cannot anticipate until it will be too late?’”

It is noteworthy that three of the five participants who did not discuss a lack of opportunities to be self-determined throughout life were recently diagnosed Autistic. These participants felt that they were only given opportunities for autonomy in their lives because they did not have an autism diagnosis growing up. For example, Nancy stated that she thinks, “as soon as that label gets slapped on you, ‘Oh, you are autistic’, then they automatically start, y’know, just those stereotypes that people expect that, ‘Oh all autistics must be the same, this is how you have to be treated.’” Similarly, Callie stated that she was “able to make choices in my day because most people aren’t aware of my autism…when people know I am Autistic it creates problems.”

Many (*n* = 13) participants also discussed how neuro-normative expectations and their fear of social censure/exclusion, led them to mask or camouflage obvious signs of their autistic traits or characteristics, despite their discomfort with doing so. For example, Alex emphatically stated that he “needs to mask to be respected … so the vast majority of us (Autistic people) always need to be masking,” and Freda reported that they “do not feel safe unless they mask.” Kawhi stated that his choice to mask or camouflage was a form of being self-determined because “choosing to mask … maintains equality and preserves an opportunity for me to show [colleagues] I can achieve.” Tia agreed that masking was a form of self-determination, but also described that masking or camouflaging lead to “feel [ing] miserable all the time” because it meant “doing things that felt wrong and bad” to her. Further complicating the issue, some of the participants described that needing to mask their feelings of distress or discomfort (for example from sensory sensitivities) often hid their subjective experience from others. While this might be protective from social disapproval in the short term, it also meant that friends, family or colleagues might not truly understand their lived experiences, which impeded their ability to gain their support in their daily lives. For example, Tia described that she masked a lot growing up and as a result, her family “did not know that I could be experiencing reality in such a different way than them. Like that just did not occur to them…so I just stopped asking for help.”

Overall, 17 of 19 participants discussed how ableist expectations and discrimination limited self-determination for Autistic people through limiting opportunities and/or decreasing one’s confidence in their ability to make appropriate choices and decisions, and in limiting their ability to present as their authentic Autistic selves. In addition to these externally imposed barriers, all participants discussed how differences with various aspects of executive processing influence their capacity to be self-determined.

#### 3.2.2. Theme 2: executive processing differences make choice and decision-making difficult

While all participants highlighted the importance and benefits of having the opportunity to make their own choices, all participants also discussed barriers to self-determination related to their perceived competence to make choices and decisions in daily life. Although, as previously discussed, some participants doubted their ability to make appropriate choices due to experiences with ableism and discrimination growing up, all participants did discuss challenges related to their executive processing abilities, which are necessary for goal-directed behavior. In particular, participants discussed challenges with emotional regulation, organization, initiation and planning, shifting attention, and flexible thinking as related to their competence to be self-determined. Of note, 11 participants were co-diagnosed with attention-deficit hyperactivity disorder (ADHD) and seven participants were co-diagnosed with anxiety disorder (five of whom were co-diagnosed with both additional diagnoses), and often attributed struggles to these co-diagnoses more than autism. However, all participants without these additional diagnoses also discussed the impact of executive processing differences on their ability to be self-determined.

Almost all participants (*n* = 16) felt that choice-making was overwhelming. In particular, 11 of these participants discussed that having “too many choices” was overwhelming. For example, related to multiple choices, James said “I have to be very careful with that. I hit ‘decision paralysis’ if I’m not careful. I tend to go into a loop in my brain over and over and over again, and have to find a way to step in and stop myself.” Similarly, Nancy said, “I do not deal well with multiple choices …. I find that I get stuck in this indecision of not being able to make a choice at all, despite the options present.” To support decision-making, Nayeli felt that she often made misguided choices because “I just pick something and hope it works because the decision-making choice is overwhelming.” In addition to stress caused by the need to make choices, several participants described how stress from other areas of life made choice-making more difficult. For example, Paul, who had to navigate university exams, stated, “… it gets worse based on stress levels as well. So, like, the more stressed I am, the more I am unable to make those basic human decisions.”

Three participants commented that making choices was difficult when there are many steps involved in a task or coming to a final decision, and they had to organize their thoughts or plan toward an informed choice. Keasik felt that “choice makes task initiation harder,” and described the process of making choices as a ‘tangled decision tree’. Using the example of a seemingly routine activity of daily living, she shared,

I live alone… I have total autonomy. It’s actually a little stressful because I have more choices than I want. Some decisions are so overwhelming, like, for example, the decision to clean my apartment. I would really like to clean my apartment, but there are a lot of things to do and where to start. It’s so overwhelming that I just never start.

Four participants discussed experiences with inflexible thinking or being so detail-focused that it interfered with decision-making. For example, Kanti reflected that their need to learn everything about the options limited their ability to make seemingly simple choices, “my obsessiveness of autism means, like, I will read for a week which kind of hand mixer is best before making a decision … I cannot just make a snap decision and asking me to gives me anxiety.” Reflecting on difficulties he has making decisions at work, Serge lamented, “Not everybody who has difficulties in a ‘gray zone’ is Autistic, but Autistic people will have challenges and count me in.” To support his ability to determine the best course of action, Serge often relied on frameworks, policies and parameters to help guide him, yet, acknowledged that this strategy could lead to further problems due to a lack of flexible thinking: “Once I [use] a framework or policy or a parameter, maybe I stick to it too much, without giving [my attention] to new information, to new possibilities.” Serge seemed to be highlighting that his executive processing differences could lead to challenges with decision-making in contexts where there may be ambiguity, uncertainty and/or conflicting factors; yet at the same time, one of his key strategies for navigating such circumstances (relying on frameworks/policies) could reinforce inflexible thinking, which in turn further hampered his ability to make the best decision. Veronica felt that she was able to make decisions, but had difficulties with changing her decisions, reflecting challenges with flexible thinking. She was fervent in her feelings that,

“once I decide something, then I appreciate it if somebody doesn’t try to change it. I’d rather know ahead of time if I need to think about it differently in order to come to my decisions about what I’m going to do. Being upfront … but it seems to me like neurotypical people put priorities opposite. I like to know what’s important first and then I can make a decision that works for me. And when I’ve come to a decision and somebody tries to make me change my mind … it’s the change part that’s hard. The change is really hard.”

Difficulties shifting focus were discussed by three participants. Both Freda and James used the term “hyperfocus” to describe their lack of choice in changing an activity. James unpacked this experience as he “hits the hyperfocus element of ADHD and autism fairly frequently …and [does not] have a choice in what I’m doing at a given time.”

Despite our participants’ unanimous agreement that they experienced challenges that decreased their opportunities and capacity to be self-determined, they offered many concrete strategies to support their and other Autistic people’s ability to be self-determined.

### 3.3. How do you want to be supported to be self-determined?

Participants discussed that self-determination does not preclude their desire or need for support from others. Rather, obtaining support on one’s terms enacts self-determination. They desired support across all three psychological needs outlined in Deci and Ryan’s (1) self-determination theory, also centered around both opportunity for and capacity to be self-determined. Their desired strategies to increase opportunity reflected (1) opportunities to make choices (autonomy), and (2) to feel supported to unmask and be valued as their authentic Autistic selves (relatedness). Participants also identified numerous strategies that (3) could provide helpful pragmatic support with executive processing (competence).

#### 3.3.1. Give me more opportunities to make choices

As indicated, a barrier to self-determination was a lack of opportunity, often due to a presumed inability to make appropriate choices. As such, many participants discussed a desire just to be given opportunities to make choices and exert autonomy, including having others adopt a presumed-competence approach, with their “choice actually listened to and validated” (Marcy).

As mentioned previously, many participants felt that decisions around food and medical autonomy were especially important. The desired strategies focused on respecting individuals’ choices based on an appreciation for their unique sensory experiences, rather than assuming a lack of capacity to make appropriate decisions. For example, Nayeli’s comment reflected the sentiment of multiple participants, when she said, “other people aren’t the ones who have to live with my sensory stuff and anxiety, [so] I need to be able to accept and reject things on my own terms.”

Many participants expressed a strong desire for others to demonstrate confidence in their ability to make choices, starting in childhood. For example, Dani discussed that as an adult, their parents have begun to “let me make decisions[they] do not always agree with my choices, [but] at this point, I’ve proven that I’m capable.” However, they went on to reflect, related to their desired leisure activities that differed from what their parent perceived they should do, “when I was a younger adult my mom definitely struggled with some of the decisions I made and was not always super supportive…she was not a fan of them, and she made it very clear that she was not a fan of them, and it became sort of a source of strife.” Therefore, participants also discussed the importance of ensuring that Autistic people, across the lifespan, are not told that their choices and desires are “wrong”,^1^ when they might just be different from what other people might have chosen.

#### 3.3.2. Support me to unmask and be valued as my authentic autistic self

Along with the desire for others to validate their choices, participants expressed a strong desire to feel respected for their choices. This feeling inherently reinforced participants’ desire to be valued as their authentic selves, including the nuances of their Autistic experience that might make them unique. Many participants expressed a desire for non-autistic people in their social and work networks to “just be comfortable with me being myself” (Stephen). Some participants, like Kanti, discussed how “neurodiverse advocacy is a passion of mine and I’ve been working toward presenting myself authentically as opposed to the homogenized acceptable version that I had been living most of my life.” However, multiple participants felt that they could not “unmask” without support and validation from others. For example, Tia stated, “I have masked for so long that I do not know how to unmask around other people. I need support learning how to even unmask.”

All participants wanted broad societal acceptance and inclusion. However, most participants discussed that, rather than waiting for broader societal change that embraces neurodiversity, they strive to or have already surrounded themselves with others who are supportive and on whom they can rely and feel emotionally safe to be their authentic selves. For example, Kanti went on to express that, in pursuit of their passion to live authentically, they “have worked very hard to build a community around myself of kind of like-minded people, so there’s a certain kind of mutual understanding, and I find a lot of acceptance in that.” Similarly, James appreciated support from “accepting others, like my family who I can rely on.”

#### 3.3.3. Pragmatic strategies for supporting executive processing differences

Even though the type of strategies that we asked about was open-ended, 13 of 19 participants articulated strategies that others could enact to accommodate their executive processing differences.

Many participants (*n* = 11) specifically identified a desire for others to scaffold choice and decision-making by providing clear and direct communication about their potential choices. For example, Freda stated that making choices is much less overwhelming with concrete support: “Pros and cons are great, like having more information about what the choices are is very helpful…[and] explanation for what happens after that,” and several participants indicated a preference for having lists that clearly outline available choices. Freda also appreciated if others could help them “categorize choices so it’s easier for me to understand and not feel overwhelmed,” providing an example of categorizing mustard at the grocery store, “if it was all just mustard all mixed together [at the grocery store], that’s overwhelming. But, if it’s ‘these are the Dijon, these are the ones that are spicy, these are the honey mustards’, that is less overwhelming.” Of note, Freda also articulated the importance of reinforcing the autonomy to make the choice to say “no,” when they articulated how they “like being explicitly told that I can make the choice because I will default to what I’m told. I do not assume that I have a choice…literally just being told that I have the option to make a choice, and that I’m safe to.”

Three participants specifically discussed that providing more time for them to come to a decision was very important. For example, Kanti expressed that “I appreciate not having to worry about a time…I’m able to make an educated decision when I can think fairly calmly, or as calm as I can get without having to worry about an extremely short time constraint.” Marcy also articulated the benefits of being given “extra” time to evaluate options:

I think that having more kind of time to process things would be really helpful because a lot of times what my first judgment call might be, is not what I end up like kind of sinking into as time goes on, and I process something. So, like having time to change my mind..and support around knowing the options, knowing that I have time to process the options, knowing that I can change my mind around the options, especially with new information coming in.

Tia’s powerful statement reflected the benefits of offering support as part of fostering autonomy, and not as a way to exert external influence or interference, “I’m afraid of asking too much of people, but I *really* want people to offer help so it does not always feel like I’m asking. I guess I’m always afraid of, like, asking too much of other people.” Offering this support without needing or expecting Autistic people to ask can help create opportunities for success, which is important to instill confidence in their ability to make appropriate decisions.

Participants frequently expressed a desire for pragmatic support, particularly regarding activities of daily living. Tia, for instance, mentioned that while she manages her own finances, her parents handle her car insurance because she dislikes “doing paperwork and filling out forms.” Many participants (*n* = 8) sought support that would provide pragmatic emotional assistance during times of overwhelm. Kawhi articulated the usefulness of having a trusted person who can articulate their needs and communicate on their behalf when they are unable to do so. Similarly, Nayeli acknowledged her tendency to feel overwhelmed by certain decisions and expressed a strong desire for someone to be physically present to discuss the matter, emphasizing the importance of an unbiased approach. She explained that by the time she seeks support for decision-making, she is often in a state of distress, and therefore requires compassion, comfort, and guidance to navigate through those moments,

definitely wanted somebody to be there in person and talk it over with and know that they don’t have an agenda about getting me to choose a certain thing … because when I ask for support about making a decision then by that time I’m, like, having a meltdown pretty much, so, first of all, they need to be able to be compassionate and comforting about that meltdown and then help me through that.

## 4. Discussion

Self-determination is a basic human right that is associated with many positive outcomes that can improve one’s quality of life. Unfortunately, research consistently shows that Autistic people experience less self-determination than non-autistic people, attributed to both decreased opportunity and capacity to be self-determined (3). Although self-determination involves causal agency, perceptions of self-determination are often based on reports from others, such as caregivers and educators, rather than the lived experiences of Autistic people (7, 30). This study aimed to (a) understand what self-determination means to Autistic people from their perspective; (b) explore their perceptions of current barriers to being self-determined, and (c) learn from Autistic people about how they would like to be supported to be self-determined.

Our participants identified that self-determination involves having influence over what happens in their lives, including both opportunities and support to make choices and decisions without unnecessary control from others. Not surprisingly, this conceptualization aligns with how self-determination is conceptualized for non-Autistic people (1–3). The foundational skills to self-determination, such as learning to make choices, express one’s preferences, make decisions and set goals are generally fostered during childhood and become more refined in adolescence, especially when people are able to increase volitional and agentic actions (31, 32). Autistic young adults identified autonomous decision-making as a key desire in their transition to adulthood, yet one that was often thwarted because they felt micromanaged and were not granted decision-making authority or had their decisions questioned when made (14). Like the participants in the study by Cheak-Zamora and colleagues (14), our participants discussed challenges with executive processes that made some aspects of self-determination difficult. However, our participants often did not receive support with these challenges even when they asked for support.

The term autonomy, a component of being self-determined, is often misinterpreted as independence. However, making one’s desires and needs known, including asking for support, *is* being autonomous (33). Like our participants, Shogren and colleagues (34) indicate the relevance of support to self-determination, as put in their succinct definition that self-determination is, “having opportunities and supports to make or cause things to happen in your life” (p. 289). Targeting support to specific areas of executive processing that interfere with choice and decision-making, while at the same time leveraging strengths in executive processes, enhances self-determination (34). However, it is notable that people who experience many other non-autism developmental diagnoses that are also associated with challenges in executive processing are often still more self-determined than Autistic people (5, 8–10, 35, 36). So, there must be something in addition to experiencing differences in executive processing that limited our participants’ opportunities to be self-determined. Autistic people are among the most discriminated and stigmatized groups of people (37–40), which we suspect is the primary reason for decreased self-determination.

Stigma involves disapproval of someone because their social identity is perceived to deviate from social norms and values in a negative way (41). It results from negative attitudes (prejudice) and behaviors (discrimination) from others. Others, such as caregivers and educators, often rate the capacity of Autistic people to be self-determined as lower than Autistic people rate themselves (13). While these ratings might be well-informed or well-intentioned, they might also reflect discrimination against the inherent ability of Autistic people to be self-determined, contributing to the lack of opportunities provided. Given the lack of perceived capacity, parents may demonstrate overprotection of their Autistic children, which has been shown to predict poorer mental health as adults (42). Internalizing prejudice related to autism can lead to self-stigma, which is also associated with poorer mental health, decreased self-esteem and self-efficacy, and behavioral responses such as a lack of initiation to pursue meaningful opportunities (18, 43). Of concern, our participants did discuss examples that might indicate self-stigma, such as concerns over making the “wrong” decisions. However, similar to other research (14), they also recognized their ability to be self-determined when given adequate support with executive processing and opportunities to exert their autonomy. Our participants advocated for a ‘presumed competence’ approach while also being offered the support they need and desire to assist with choice and decision-making. These findings align with Webster and Garvis, whose participants appreciated a presumed competence approach to work out solutions independently, develop self-determination and feel successful (30). However, our findings also counter those of Webster and Garvis, whose participants felt most successful when they were able to act without support from others (30).

Autistic people use masking and selective disclosure to manage the impact of stigma (18). However, masking is associated with numerous negative outcomes for Autistic people, and while it can be an asset to decrease prejudice and discrimination, it can also reaffirm the stigma of being Autistic because it is used to hide ‘flawed’ or ‘faulty’ characteristics (44). Our participants used masking as a strategy to counteract stigma and enhance opportunities to be self-determined. Yet, they also discussed the negative implications of masking on their wellbeing and a desire to live in a society where they could “unmask” and be authentic. Our participants’ desire to unmask differs from previous research in which Autistic men felt that learning to act in neurotypical ways (to mask) was a positive experience that enhanced autonomy, especially when they were “late-diagnosed” and had increased opportunities for this practice (30). Interestingly, our participants who felt that they were the most self-determined were also often “late-diagnosed.” However, rather than talking positively about opportunities to develop neurotypical behaviors, they felt that they were more self-determined because they did not experience prejudice and discrimination associated with the label.

Consistent with other research (45), some of our participants felt that they were only given respect for their choices and decisions and opportunities to be self-determined because they did not disclose their diagnoses of autism to others. Their efforts, which reinforced ableist discourses that Autistic people should strive to “pass as normal,” likely took away their autonomy to make decisions aligned with their Autistic identity. Parents, educators, professionals and others in general society should support Autistic individuals to become more self-determined by exploring positive aspects of their authentic Autistic identity, building on their strengths, respecting their desires and choices, and providing the support and opportunities they need to be successful in the choices and decisions they make (18, 46). Societal level and systemic changes aimed at reinforcing anti-ableist practices and policies are also necessary to broadly counteract autism stigma and enable Autistic people to feel safe to unmask. Empowering Autistic people as key consultants to ensure that these initiatives align with their needs and priorities is necessary and critical to their success (47).

### 4.1. Limitations

As with any research, our findings will not represent the experiences of all Autistic people. However, we do take comfort that our team engaged six Autistic (and two non-autistic) researchers throughout the research process. We believe that this team composition is fundamental given evidence that the lived experience of being autistic may offer unique insights and perspectives into our data that a non-autistic person might not perceive (48, 49). We also recognize that our participants learned about the study through electronic means of recruitment; therefore, Autistic persons without internet access were likely not represented in our study findings. Additionally, we did not ask about the cultural background of our participants, but we do know that they all lived in North America at the time of data collection. Culture can influence the degree to which people are supported to be self-determined (50). Therefore, our findings may not apply to Autistic people from cultures outside of those represented by our participants.

Our questions focused more on autonomy (choice-making) than the other psychological needs outlined in self-determination theory (competence, relatedness). Although our analyses included rich findings related to all areas, we acknowledge that the questions we asked may have swayed the results we found toward autonomy.

### 4.2. Directions for future research

Research specific to self-determination experiences for Autistic people, and especially based on the perspective of Autistic people, is relatively limited (7). Furthermore, research that garners the perspective of Autistic people with co-occurring intellectual disability and/or those who do or prefer to communicate in non-speaking ways is almost non-existent (51). Therefore, we strongly advocate for research that continues to garner perspectives on and desired support to be, self-determined from the perspective of Autistic people, and especially people with co-occurring intellectual disability and/or those who communicate in a variety of ways.

Although we garnered perspectives of people who felt varying degrees of self-determination, all of our participants felt thwarted to some extent. Research with Autistic people who feel a strong sense of self-determination is vital to provide a fulsome perspective on those experiences and insight into potential influences on positive experiences. This research could inform potential strategies and supports to enhance experiences for Autistic people more broadly.

Finally, given our perspective that stigma is a (the) major contributor to thwarted opportunities and adequate support for self-determination for Autistic adults without intellectual disability, there is a crucial need for the continued development and evaluation of anti-stigma initiatives related to autism. Furthermore, we advocate for the development and evaluation of these initiatives for Autistic people across the variability of intellectual ability and support needs. Anti-stigma interventions, such as autism-friendly spaces, increased inclusive media representation, and education training tools, do exist (16), but clearly, more work is needed given the ongoing pervasiveness of autism stigma.

## 5. Conclusion

Self-determination holds the same significance for autistic individuals as for their non-autistic counterparts. It encompasses making choices and decisions free from undue external influences. However, Autistic people live less self-determined lives than others, due to challenges posed by executive processing differences, limited opportunities and discrimination, influenced by stigma and ableist expectations.

Autistic adults desire to be self-determined and can flourish with support, as they determine to be appropriate, which might look different from support commonly offered or sought by non-autistic people. Autistic individuals desire support that respects their choices, validates their authentic selves, and provides pragmatic assistance, particularly in managing executive processing differences. Although individualized supports were discussed, the ideal desired support was for an inclusive society that values and respects their neurodivergence, rather than imposing ableist expectations. An inclusive society can only be achieved by reducing stigma and discrimination against Autistic people. Overall, addressing barriers at all levels, including societal-level approaches, and offering appropriate support can promote self-determination and empower autistic individuals in all aspects of their lives.

## Data availability statement

The original contributions presented in the study are included in the article/supplementary material, further inquiries can be directed to the corresponding author.

## Ethics statement

The studies involving humans were approved by Research Ethics Board, University of Alberta. The studies were conducted in accordance with the local legislation and institutional requirements. The participants provided their written informed consent to participate in this study.

## Author contributions

ST-H contributed to all aspects of the study, including conception, design, data collection, analysis, interpretation and drafting of the manuscript. JR and HB contributed to conception, design, analysis, interpretation, and critical review of the manuscript. EC and AX contributed to data collection, analysis, interpretation, and critical review of the manuscript. CD, AL, AB, and AK contributed to design, analysis, interpretation, and critical review of the manuscript. All authors approved the submitted manuscript and agreed to be accountable for this work.

## Funding

This study was funded by a Social Sciences and Humanities Research Council of Canada (SSHRC) Partnership Development Grant (890-202-0012; PI: Sandy Thompson-Hodgetts).

## Conflict of interest

The authors declare that the research was conducted in the absence of any commercial or financial relationships that could be construed as a potential conflict of interest.

## Publisher’s note

All claims expressed in this article are solely those of the authors and do not necessarily represent those of their affiliated organizations, or those of the publisher, the editors and the reviewers. Any product that may be evaluated in this article, or claim that may be made by its manufacturer, is not guaranteed or endorsed by the publisher.

## Footnotes

^1^Our participants discussed experiences with daily choices, such as leisure (e.g., not wanting to participate in a sport) and self-care (e.g., food preferences) activities. We acknowledge that exerting parental control to try to keep one’s children safe and healthy may be required by parents.

## References

[1] Ryan RM Deci EL. Self-determination theory: basic psychological needs in motivation, development, and wellness. New York: The Guildford Press (2017).

[2] Shogren KA Wehmeyer ML Palmer SB Rifenbark GG Little TD. Relationships between self-determination and postschool outcomes for youth with disabilities. J Spec Edu. (2015) 48:256–67. doi: 10.1177/0022466913489733

[3] Wehmeyer ML Shogren KA Little TD Lopez SJ. Development of self-determination through the life-course. Netherlands: Springer (2017).

[4] United Nations. Convention on the rights of persons with disabilities. Treaty Series, 2515, 3. (2006). 37 p.

[5] Weiss JA Riosa PB. Thriving in youth with autism spectrum disorder and intellectual disability. J Autism Dev Disord. (2015) 45:2474–86. doi: 10.1007/s10803-015-2412-y, PMID: 25772536 PMC4513199

[6] Denney SC Daviso AW. Self-determination: a critical component of education. Am Second Educ. (2012) 40:43–51.

[7] White K Flanagan TD Nadig A. Examining the relationship between self-determination and quality of life in young adults with autism spectrum disorder. J Dev Phys Disabil. (2018) 30:735–54. doi: 10.1007/s10882-018-9616-y

[8] Chou YC Wehmeyer ML Palmer SB Lee J. Comparisons of self-determination among students with autism, intellectual disability, and learning disabilities: a multivariate analysis. Focus Autism Dev Dis. (2017) 32:124–32. doi: 10.1177/1088357615625059

[9] Hodgetts S Richards K Park E. Preparing for the future: multi-stakeholder perspectives on autonomous goal setting for adolescents with autism spectrum disorders. Disabil Rehabil. (2018) 40:2372–9. doi: 10.1080/09638288.2017.1334836, PMID: 28592161

[10] Qian X Shogren K Odejimi OA Little T. Differences in self-determination across disability categories: findings from national longitudinal transition study 2012. J Disabil Policy Stu. (2022) 32:245–56. doi: 10.1177/1044207320964396

[11] Tomaszewski B Kingler LG Pugliese CE. Self-determination in autistic transition-aged youth without intellectual disability. J Autism Dev Disord. (2022) 52:4067–78. doi: 10.1007/s10803-021-05280-6, PMID: 34536165 PMC8930783

[12] Cheak-Zamora NC Maurer-Batjer A Malow BA Coleman A. Self-determination in young adults with autism spectrum disorder. Autism. (2020) 24:605–16. doi: 10.1177/1362361319877329, PMID: 31561711

[13] Tomaszewski B Kraemer B Steinbrenner JR Smith DaWalt L Hall LJ Hume K. Student, educator, and parent perspectives of self-determination in high school students with autism spectrum disorder. Autism Res. (2020) 13:2164–76. doi: 10.1002/aur.2337, PMID: 32743977 PMC7781162

[14] Cheak-Zamora N Tait A Coleman A. Assessing and promoting independence in young adults with autism spectrum disorder. J Dev Behav Pediatr. (2022) 443:130–9. doi: 10.1097/DBP.0000000000001021 34636359

[15] Morán ML Hagiwara M Raley SK Alsaeed AH Shogren KA Qian X. Self-determination of students with autism Spectrum disorder: a systematic review. J Dev Phys Disabil. (2021) 33:887–908. doi: 10.1007/s10882-020-09779-1, PMID: 37540792

[16] Turnock A Langley K Jones CRJ. Understanding stigma in autism: a narrative review and theoretical model. Autism Adulthood. (2022) 4:76–91. doi: 10.1089/aut.2021.0005, PMID: 36605561 PMC8992913

[17] Hedges SH Kirby AV Sreckovic MA Kucharczyk S Hume K Pace S. “Falling through the cracks”: challenges for high school students with autism spectrum disorder. High Sch J. (2014) 2014:64–82. doi: 10.1353/hsj.2014.0014

[18] Han E Scior K Avramides K Crane L. A systematic review on autistic people's experiences of stigma and coping strategies. Autism Res. (2022) 15:12–26. doi: 10.1002/aur.2652, PMID: 34881514

[19] Markula P Silk M. Qualitative research for physical culture. Basingstoke, UK: Palgrave Macmillan (2011).

[20] Shakespeare T. The social model of disability In: Davis LJ, editor. The disability studies reader. 4th ed. New York: Routledge (2013). 214–21.

[21] Fletcher-Watson S Adams J Brook K Charman T Crane L Cusack J. Making the future together: shaping autism research through meaningful participation. Autism. (2018):943–53. doi: 10.1177/1362361318786721 30095277 PMC6512245

[22] Nicolaidis C Raymaker D McDonald K Dern S Ashkenazy E Boisclair C. Collaboration strategies in nontraditional community-based participatory research partnerships: lessons from an academic−community partnership with autistic self-advocates. Prog Comm Hlth Partn. (2011) 5:143. doi: 10.1353/cpr.2011.0022 PMC3319698 21623016

[23] Botha M Cage E. Autism research in crisis: a mixed methods study of researcher’s constructions of autistic people and autism research. Front Psychol. (2022) 13:1050897. doi: 10.3389/fpsyg.2022.1050897 36506950 PMC9730396

[24] Hacker K. Community-based participatory research. Thousand Oaks, CA: SAGE Publications Ltd. (2017). 139 p.

[25] Levitt HM Motulsky SL Wertz FJ Morrow SL Ponterotto JG. Recommendations for designing and reviewing qualitative research in psychology: promoting methodological integrity. Qual Psychol. (2017) 4:2–22. doi: 10.1037/qup0000082

[26] Vasileiou K Barnett J Thorpe S Young T. Characterising and justifying sample size sufficiency in interview-based studies: systematic analysis of qualitative health research over a 15-year period. BMC Med Res Methodol. (2018) 18:148. doi: 10.1186/s12874-018-0594-7 30463515 PMC6249736

[27] Braun V Clarke V. Reflexive thematic analysis: a practical guide. London: SAGE (2021).

[28] Davies M. Concept mapping, mind mapping and argument mapping: what are the differences and do they matter? High Educ. (2011) 62:279–301. doi: 10.1007/s10734-010-9387-6

[29] Charmaz K. Constructing grounded theory. London: Sage (2006). 208 p.

[30] Webster AA Garvis S. What does success mean for autistic men? A narrative exploration of self-determination. Autism Dev Lang Impair. (2020) 5:1–13. doi: 10.1177/2396941520945522 36381554 PMC9620464

[31] Palmer SB Wehmeyer ML Shogren KA. The development of self-determination during childhood In: Wehmeyer ML Shogren KA Little TD Lopez SJ, editors. Development of self-determination through the life-course. Dordrecht: Springer (2017). 71–88.

[32] Wehmeyer ML Shogren KA. The development of self-determination during adolescence In: Wehmeyer ML Shogren KA Little TD Lopez SJ, editors. Development of self-determination through the life-course. Dordrecht: Springer (2017). 89–98.

[33] Wehmeyer ML Shogren KA. Applications of the self-determination construct to disability In: Wehmeyer ML Shogren KA Little TD Lopez SJ, editors. Development of self-determination through the life-course. Dordrecht: Springer (2017). 111–23.

[34] Shogren KA Mosconi MW Raley SK Dean EE Edwards B Wallisch A. Advancing the personalization of assessment and intervention in autistic adolescents and young adults by targeting self-determination and executive processes. Autism Adulthood. (2021) 3:289–99. doi: 10.1089/aut.2021.0010 36601638 PMC8992922

[35] Rogers M Tannock R. Are classrooms meeting the basic psychological needs of children with ADHD symptoms? A self-determination theory perspective. J Atten Disord. (2018) 22:1354–60. doi: 10.1177/1087054713508926, PMID: 24327276

[36] Spaniol M Danielsson H. A meta-analysis of the executive function components inhibition, shifting, and attention in intellectual disabilities. J Intellect Disabil Res. (2022) 66:9–31. doi: 10.1111/jir.12878 34498787

[37] Cooper KE Smit L Russell A. Social identity, self-esteem, and mental health in autism. Eur J Soc Psychol. (2017) 2017:844–54. doi: 10.1002/ejsp.2297

[38] Khudiakova V Chasteen AL. The experiences of stigmatization and discrimination in autistic people of different genders and sexualities. J Interpers Relat Intergroup Relat Identity. (2022) 2022:15. doi: 10.33921/isnu8742

[39] Mazumder R Thompson-Hodgetts S. Stigmatization of children and adolescents with autism Spectrum disorders and their families: a scoping study. Rev J Autism Dev Disord. (2019) 6:96–107. doi: 10.1007/s40489-018-00156-5

[40] Pearson A Rose K. A conceptual analysis of autistic masking: understanding the narrative of stigma and the illusion of choice. Autism Adulthood. (2021) 3:52–60. doi: 10.1089/aut.2020.0043, PMID: 36601266 PMC8992880

[41] Goffman E. Stigma: Notes on the management of spoiled identity. Eaglewood Cliffs: Pretence Hall (1967). 168 p.

[42] Lee JYS Whittingham K Mitchell AE. Childhood experiences of being parented, adult attachment, psychological inflexibility, social engagement, and mental health of autistic adults. Res Dev Disabil. (2022) 130:104343. doi: 10.1016/j.ridd.2022.104343, PMID: 36152473

[43] Corrigan PW Watson AC. Understanding the impact of stigma on people with mental illness. World Psychiatry. (2002) 1:16–20. PMID: 16946807 PMC1489832

[44] Schneid I Raz AE. The mask of autism: social camouflaging and impression management as copying/normalization from the perspectives of autistic adults. Soc Sci Med. (2020) 248:112826. doi: 10.1016/j.socscimed.2020.112826, PMID: 32036269

[45] Thompson-Hodgetts S Labonte C Mazumder R Phelan S. Helpful or harmful? A scoping review of perceptions and outcomes of autism diagnostic disclosure to others. Res Autism Spect Dis. (2020) 77:101598. doi: 10.1016/j.rasd.2020.101598, PMID: 37640496

[46] Brown HM Stahmer AC Dwyer P Rivera S. Changing the story: how diagnosticians can support a neurodiversity perspective from the start. Autism. (2021) 25:1171–4. doi: 10.1177/13623613211001012, PMID: 34232104

[47] Collis RB. Over and over and over: a continued call for autistic voices. The Canadian J Autism Equity. (2021) 1:18–22. doi: 10.15173/cjae.v1i1.4984

[48] Finch TL Mackintosh J Petrou A McConachie H Le Couteur A Garland D. “We couldn’t think in the box if we tried. We can’t even find the damn box”: a qualitative study of the lived experiences of autistic adults and relatives of autistic adults. PLoS One. (2022) 17:e0264932. doi: 10.1371/journal.pone.0264932, PMID: 35286347 PMC8920184

[49] Gillespie-Lynch K Kapp SK Brooks PJ Pickens J Schwartzman B. Whose expertise is it? Evidence for autistic adults as critical autism experts. Front Psychol. (2017) 8:article 438. doi: 10.3389/fpsyg.2017.00438 28400742 PMC5368186

[50] Sheldon KM. The self-determination theory perspective on positive mental health across cultures. World Psychiatry. (2012) 11:101–2. doi: 10.1016/j.wpsyc.2012.05.017, PMID: 22654936 PMC3363380

[51] Nicholas DB Orjasaeter JD Zwaigenbaum L. Considering methodological accommodation to the diversity of ASD: a realist synthesis review of data collection methods for examining first-person experiences. Rev J Autism Dev Disord. (2019) 6:216–32. doi: 10.1007/s40489-019-00164-z

